# The cross-cultural adaptation and psychometric evaluation of a Chinese version of the postoperative symptom severity (PoSSe) scale

**DOI:** 10.1038/s41405-025-00333-9

**Published:** 2025-05-22

**Authors:** Xiang Li, Zefan Niu, Chen Gao, Annika Kroeger, Georgios Tsakos, Bolong Li, Jiaqi Zhu, Gang Chen, Thomas Dietrich

**Affiliations:** 1 Department of Dentistry, School of Health Sciences, College of Medicine and Health, University of Birmingham, Birmingham, B5 7EG UK; 2 Department of Oral and Maxillofacial Surgery, Hospital and School of Stomatology, Tianjin Medical University, Tianjin, 300070 China; 3 Department of Oral Surgery, Birmingham Dental Hospital, Birmingham Community Healthcare NHS Foundation Trust, Birmingham, B5 7EG UK; 4 Department of Epidemiology and Public Health, University College London, London, WC1E 7HB UK

**Keywords:** Third molar removal, Oral-health-related quality of life

## Abstract

**Background:**

The postoperative symptom severity (PoSSe) scale, which was developed in the UK, measures the impact of postoperative morbidity on patients’ quality of life after lower third molar surgery. It has recently been used in Chinese populations but without having been adapted and validated for these populations. The aim of this study was to cross-culturally adapt and psychometrically evaluate a Chinese version (Simplified Chinese) of the PoSSe scale for applications in third molar surgery in Chinese patient populations.

**Methods:**

We employed a rigorous multi-step cross-cultural adaptation process, including forward and backward translation followed by pilot testing, where participants documented the relevance and ease of understanding of the PoSSe items. The psychometric evaluation of the final Chinese version took place in a sample of 101 patients undergoing lower third molar surgery in Tianjin, China. Cronbach’s Alpha (*α*) coefficient was calculated for the reliability evaluation, while the Spearman correlation coefficient (*r*_*s*_) and Pearson’s correlation coefficient (*r*) were used for validity assessment.

**Results:**

The PoSSe scale demonstrated excellent internal consistency (Cronbach’s *α *= 0.80 for the whole sample; *α *= 0.80 among patients with bone removal during surgery; *α* = 0.81 among patients without bone removal during surgery). For validity assessment, PoSSe scores had statistically significant associations with the extent of surgical trauma (osteotomy and duration of surgery), self-reported pain and clinically assessed trismus. The strength of these associations varied between the two groups (with and without bone removal during surgery) in the expected direction. The results suggest that the Chinese version of the PoSSe scale has acceptable linguistic clarity, cultural relevance, and context appropriateness, showing excellent internal consistency and validity and can be confidently used for clinical and research applications in Chinese patient populations.

**Conclusions:**

The PoSSe scale has been successfully cross-culturally adapted for postoperative use among Chinese patients undergoing third molar surgery and demonstrated successful psychometric assessment for its reliability and validity, which allows future informative studies in China, also in terms of comparison across countries involving China that could assess the cultural equivalence of the measure.

## Introduction

The surgical extraction of lower third molars (LM3) is one of the most widely performed operations in the field of oral surgery. The procedure is associated with significant postoperative morbidity, including pain, trismus, swelling, and risk of alveolar osteitis, which adversely affect patients’ quality of life (QoL). Patient reported outcome measures (PROMs) are increasingly relevant in clinical research, and quality of life is therefore an important outcome in clinical studies that aim to minimise postoperative morbidity following LM3 surgery. In order to measure the postoperative quality of life of patients, the postoperative symptom severity (PoSSe) scale was developed in the UK and published in 2001 [1]. The scale has since been applied in several studies in English-speaking countries over the past decades [2–5].

The PoSSe scale has also been used in studies in Chinese populations in recent years [6–8]. It appears that these applications were based on ad-hoc translations of the original PoSSe scale from English to Chinese by the respective study investigators. However, using an instrument developed in a different language ideally requires a methodologically robust approach of cross-cultural adaptation to ensure that the translated version accurately reflects not only the linguistic but also cultural concepts measured in the original instrument. This seems pertinent in order to be able to use the PoSSe scale in clinical research or practice in China, given the differences in culture and languages, as well as healthcare systems and clinical procedures between China and the UK. A rigorously developed and validated, cross-culturally adapted Chinese version of the PoSSe scale would be highly desirable as a clinical and research tool to ensure valid, consistent, and comparable assessment of this important endpoint in clinical practice and research.

Therefore, the purpose of the work presented here was to cross-culturally adapt a Simplified Chinese version of the PoSSe scale and psychometrically evaluate this version for its applications in third molar surgery in Chinese patient populations.

## Methods

### Study design and procedures

We produced the PoSSe scale in Simplified Chinese and evaluated its performance by following a four-step process, including (1) forward translation, (2) backward translation, (3) pilot testing in a small sample, and (4) psychometric testing, as described by Guillemin et al. and Acquadro et al. [9, 10].

The study was approved by the Ethical Review Committee at the Hospital and School of Stomatology, Tianjin Medical University, Tianjin, China (AER number: TMUhMEC20211222).

#### Translation steps: forward translation and backward translation

The original PoSSe scale was firstly translated into Simplified Chinese by two independent forward translators. The forward translators were native Chinese speakers who were proficient in English, were oral surgeons, and were unfamiliar with the PoSSe scale. A discussion panel was then organised by study investigators, during which disagreements on translation were addressed via discussion between translators, forming a universal forward version of the PoSSe scale (hereafter called forward version). A forward translation report was drafted for recording differences in translation and corresponding reasons. The forward version was subsequently independently backward translated to English by two different translators who were professional medical translators. A backward version was obtained after a consensus was reached between backward translators and a backward translation report was recorded. The backward version was then compared to the original scale in order to identify potential problems with the forward translation, which was then amended to form a pilot-test version for further evaluation.

#### Pilot testing in a small sample

The purpose of this step was to assess the linguistic clarity, cultural relevance, and context appropriateness of the pilot-test version of the scale in a small group of study volunteers recruited from the Hospital and School of Stomatology, Tianjin Medical University, Tianjin, China. The inclusion criteria were: (1) written informed consent obtained; (2) more than 15 years old; (3) got one impacted LM3 (position A, B, or C, and class I or II type of impaction according to Pell and Gregory classification) extracted at a single surgical visit. Volunteers were excluded from the study if they (1) had any systemic disease; (2) had presence of other pathology in relation to lower third molar, such as pericoronitis, cysts, or tumours; (3) withdrew consent or could not provide any data during the follow-up period.

Further adjustment of the pilot-test version was implemented according to the feedback from volunteers, and linguistic improvement was done by study investigators after the adjustment, then forming a final version of the PoSSe scale in Simplified Chinese (hereafter called final version).

#### Psychometric testing

This step involved the evaluation of the reliability and validity of the final version obtained in the pilot testing among a larger sample of the target population. Study participants were recruited from the Hospital and School of Stomatology, Tianjin Medical University, during the period from  5 March 2022 to 30 April 2023. The inclusion and exclusion criteria were identical to those used for the pilot testing described above. Study volunteers participating in the pilot testing mentioned above were not enrolled in the psychometric testing.

All participants gave written informed consent for study participation before surgery. The preoperative data were collected at the surgery visit (T0). This included a photograph of the patients’ mouth at maximum opening taken by the study investigators using mobile phones with a reference frame as previously described by Xiang et al. [11]. The LM3 surgery was then performed following standard clinical procedures, during which surgical data were recorded by study investigators. These included surgery duration (the period from the beginning of incision to the end of suturing), requirement of flap surgery (yes/no), amount of bone removal measured using a 4-point Likert scale (none, minor, moderate, severe), tooth sectioning (yes/no), alveolar filling (yes/no), and drainage (yes/no). After surgery, the postoperative prescriptions, including antibiotics, analgesics, and steroids, were recorded by study investigators. Study participants were instructed to record postoperative data on pain, analgesic consumption, and the intake of antibiotics and steroids in a diary, where the name, administration frequency, and total amount of daily intake of these medications were self-recorded by the patients. Pain was measured by using a 100-mm Visual Analogue Scale (VAS), whereby patients were asked to rate their average pain on each postoperative follow-up day (T1 – T7). In addition, patients were asked to take photographs of maximal mouth opening on postoperative day 1 (T1), using the same method as investigators performed preoperatively. Study investigators contacted participants on T1 to remind patients to take and upload/send the photographs for determination of trismus. Pre- and post-operative mouth opening was determined from photographs via the method proposed by Xiang et al. [11], using ImageJ Software [12] (Version 1.53s on the macOS platform). Patients attended the clinic 1 week after surgery (T7), where diaries were collected, and patients were asked to complete the final version of the PoSSe scale. If patients did not attend the final visit, an electronic data collection form would be offered and collected online.

All data and photographs were uploaded onto an electronic database system (REDCap) hosted at the University of Birmingham [13, 14].

### Statistical analyses

#### Sample size

For the small sample test, a group of 10–20 participants was deemed sufficient as suggested by Fayers et al. and Francis et al. [15, 16]. A sample of 100 study participants is recommended for the assessment of internal reliability and validity in a cross-sectional design [15, 17, 18].

#### Statistical methods

Data were analysed using STATA SE 17. Summary statistics were calculated as appropriate. We conducted complete case analyses, i.e., values missing for any reason were not imputed. As we expected impacts to be associated with the amount of surgical trauma, statistical analyses were performed for the whole study sample as well as for patients who did or did not require bone removal separately. Trismus (mouth opening) was calculated in three ways: (1) absolute values of postoperative mouth opening in mm, (2) absolute reduction over baseline (T1-baseline) in mm, and (3) percent relative reduction [(T1-baseline) *100/baseline]. To evaluate internal consistency reliability of the PoSSe scale, Cronbach’s Alpha (*α*) coefficient was calculated. The validity assessment was achieved by correlational analyses, using the Spearman correlation coefficient (*r*_*s*_) and Pearson’s correlation coefficient (*r*), respectively. The former was calculated to evaluate the association between PoSSe score and postoperative pain (on each postoperative day and using a mean score) as well as the surgery duration, while the latter was used for correlational analyses between PoSSe score and trismus (mouth opening). The normality of data for validity assessment was checked using quantile plots. All statistical tests were two-sided at α = 0.05.

## Results

### Translation steps: forward translation and backward translation

Two independent translation drafts were obtained from independent forward translators. 13 minor translation differences were observed between the two versions (supplementary Table. 1). A universal forward translation version of the PoSSe scale was formed, following corrections and amendments as agreed by forward translators (supplementary materials. 1). Similarly, there were 14 differences between the two independent backward translation versions (supplementary table. 2), and the consensus backward version was agreed by discussion (supplementary materials 2).

Comparing the consensus backward version with the original text, 21 translation differences were found. Most of these were judged as being minor and not requiring changes to the forward version (supplementary Table 3a). However, four differences led to minor adjustments to the forward version (supplementary Table 3b). Following preliminarily linguistic improvement, a pilot-test version of PoSSe scale in Chinese was formed (supplementary materials 3).

### Pilot testing in a small sample

15 volunteers (7 males, 8 females) with an age range of 20–36 years were recruited for the pilot testing. From the perspective of linguistic clarity, feedback from the volunteers indicated that the questions and options in the pilot-test PoSSe scale were easy to understand and made sense in the context of the surgery and recovery experience.

However, participants noted two issues with the answer options to question 10 (not exhaustive) and question 12 (inconsistent with the question asked) in the original scale. Specifically, for question 10, there was no appropriate option to select for patients who experienced some degree of postoperative pain but did not take any analgesics. The description of option A was therefore modified from “I have had no pain” to “I have had no pain or did not take analgesics”. Additionally, option B of question 12 was changed from “One day” to “Once”. No further issues were identified during pilot testing.

**Fig. 1:**
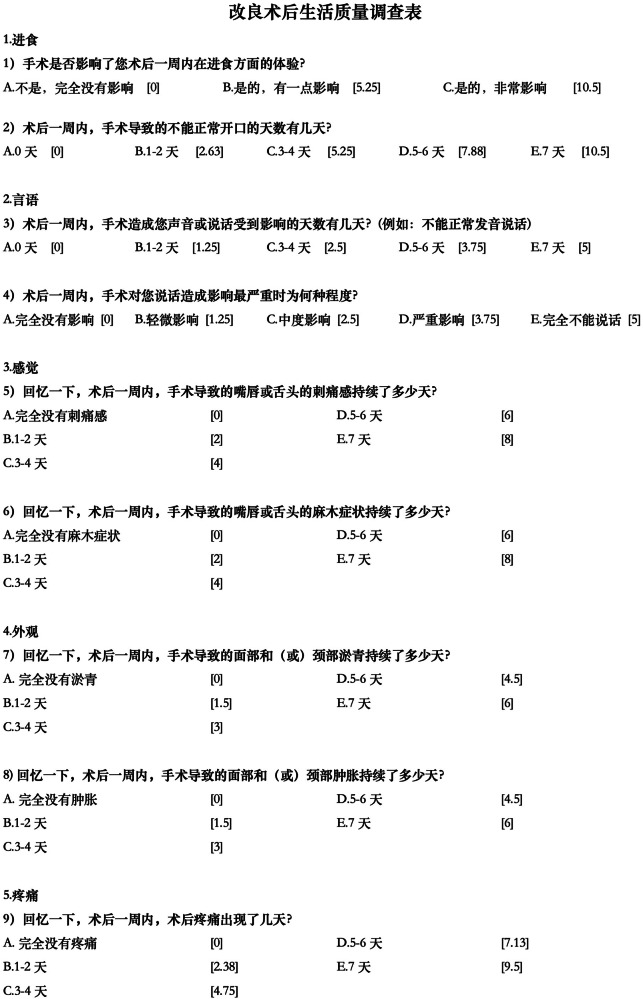

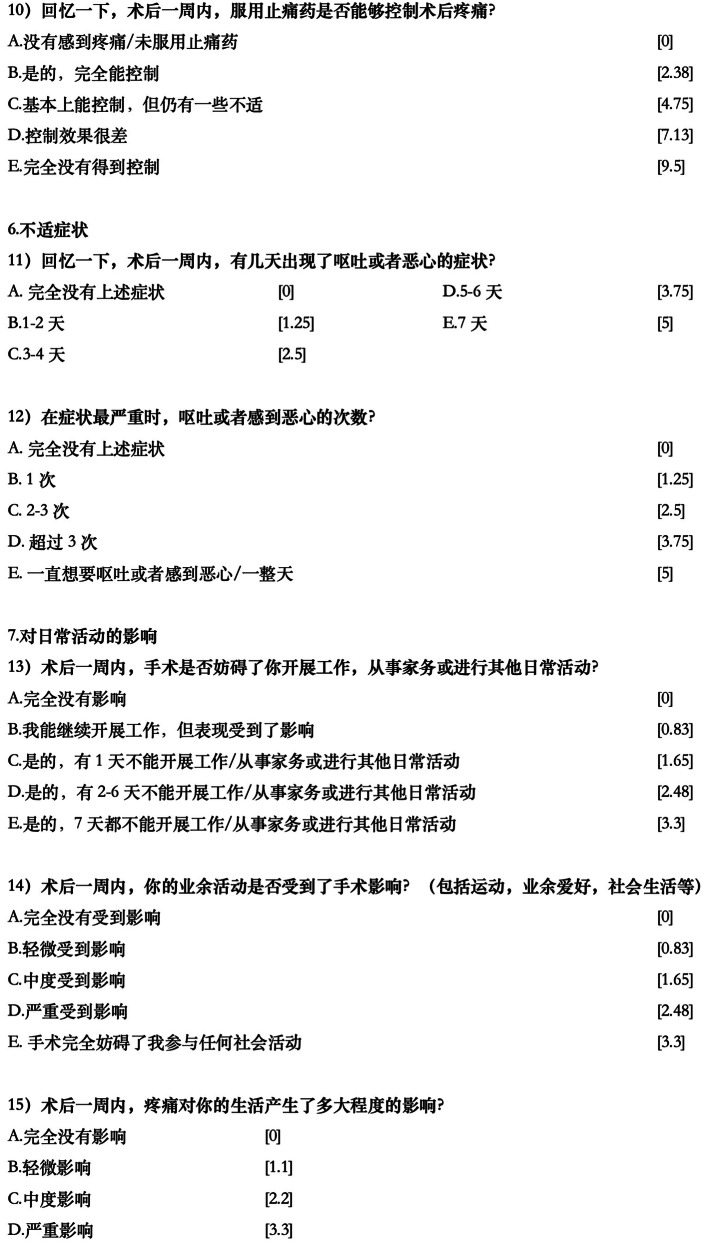
Final Chinese version of the PoSSe scale.

A few further minor linguistic improvements were made by the study investigators so that the Chinese statement would match better with the habits of language expressions in daily communication, resulting in the final version (Fig. 1).

### Psychometric testing

#### Sample characteristics

119 patients with a mean age of 25.5 years (42 male, 77 female) met the inclusion criteria and were recruited in the study (Table 1). 55 patients did not require bone removal (NB group), whereas 64 had various degrees of bone removal (FB group) during the surgery.

**Table 1:** Descriptive statistics of demographic information and perioperative data.

Items/Groups	N^a^	Overall	*n*	No bone removal	*n*	Bone removal
**Age (y, mean±SD)**	119	25.5 ± 5.1	55	25.1 ± 5.1	64	25.8 ± 5.2
**Sex**	119		55		64	
Male		42 (35.3%)		17 (30.9%)		25 (39.1%)
Female		77 (64.7%)		38 (69.1%)		39 (60.9%)
**Seniority of clinicians**	119		55		64	
Senior oral surgeon		85 (71.4%)		31 (56.4%)		54 (84.4%)
Specialty trainee		34 (28.6%)		24 (43.6%)		10 (15.6%)
**Tooth sectioning**	119		55		64	
Yes		88 (74.0%)		26 (47.3%)		62 (96.9%)
No		31 (26.0%)		29 (52.7%)		2 (3.1%)
**Socket filling**	119		55		64	
Yes		75 (63.0%)		35 (63.6%)		40 (62.5%)
No		44 (37.0%)		20 (36.4%)		24 (37.5%)
**Drainage**	119		55		64	
Yes		5 (4.2%)		1 (1.8%)		4 (6.3%)
No		114 (95.8%)		54 (98.2%)		60 (93.7%)
**Surgery duration (min)**	119	18.3 ± 10.1	55	13.0 ± 6.9	64	22.8 ± 10.4
**VAS score (mean±SD)**						
T 1	99	32.1 ± 25.8	43	33.8 ± 28.1	56	30.7 ± 24.2
T 2	99	24.7 ± 23.2	43	26.3 ± 24.3	56	23.4 ± 22.6
T 3	98	18.6 ± 21.0	42	21.3 ± 24.2	56	16.6 ± 18.2
T 4	98	14.3 ± 19.0	42	15.1 ± 21.7	56	13.8 ± 16.9
T 5	98	11.4 ± 18.2	42	11.6 ± 20.5	56	11.2 ± 16.3
T 6	98	8.3 ± 16.0	42	8.4 ± 18.3	56	8.2 ± 14.1
T 7	98	5.7 ± 11.8	42	5.8 ± 13.8	56	5.7 ± 10.2
Mean	99	16.8 ± 16.6	43	18.4 ± 19.6	56	15.7 ± 14.0
**Mouth opening**						
Preoperatively, T0(mm)	109	38.7 ± 7.9	51	38.4 ± 8.3	58	39.1 ± 7.5
Postoperatively, T1(mm)	90	27.2 ± 12.0	40	29.7 ± 11.5	50	25.3 ± 12.1
Absolute reduction (mm)	87	−11.2 ± 12.0	40	−8.0 ± 11.5	47	−13.8 ± 11.9
Relative reduction (%)	87	−28.3% ± 29.9%	40	−20.2% ± 29.1%	47	−35.3% ± 29.1%
**PoSSe score (mean±SD)**	101	25.1 ± 10.4	44	22.0 ± 10.2	57	27.5 ± 10.0
**Analgesics** (***n***, **%**)						
Post-prescribed	119	74 (62.1%)	55	34 (61.8%)	64	40 (62.5%)
Actual consumption^b^						
T 1	99	60 (60.6%)	43	21 (48.4%)	56	39 (69.6%)
T 2	99	45 (45.5%)	43	17 (39.5%)	56	28 (50.0%)
T 3	98	35 (35.7%)	42	15 (35.7%)	56	20 (35.7%)
T 4	97	20 (20.6%)	41	10 (24.4%)	56	10 (17.9%)
T 5	97	13 (13.4%)	42	2 (4.8%)	55	11 (20.0%)
T 6	98	12 (12.2%)	42	2 (4.8%)	56	10 (17.9%)
T 7	98	5 (5.1%)	42	0	56	5 (8.9%)
**Antibiotics** (***n***, **%)**						
Post-prescribed	119	101 (84.9%)	55	46 (83.6%)	64	55 (85.9%)
Actual consumption^b^	97	85 (87.6%)	42	35 (83.3%)	55	50 (90.9%)
**Steroids** (***n***, **%**)						
Post-prescribed	119	23 (19.3%)	55	4 (7.3%)	64	19 (29.7%)
Actual consumption^b^	96	21 (21.9%)	42	2 (4.8%)	54	19 (35.2%)

^a^The number of patients varies due to missing values.

^b^The number includes patients who took medications either prescribed by study investigators or prepared by themselves.

More than 70% of all surgeries were performed by senior oral surgeons (specialists with more than 10 years of clinical working experience in Oral Surgery). The proportions of surgery performed by senior oral surgeons in subgroups were 56.4% (NB) and 84.4% (FB), respectively. The remaining cases were conducted by specialty trainees in Oral Surgery.

During the surgeries, 47.3% of cases in the NB group required tooth sectioning, compared to 96.9% in the FB group. A haemostatic collagen sponge was applied in approximately 63% of patients in both groups at operators’ discretion. Five patients received drainages (1 in the NB group and 4 in the FB group). On average, surgery duration was higher in the FB group (22.8 min) than in the NB group (13.0 min).

The worst postoperative pain occurred on the first postoperative day (T1) and VAS scores declined steadily over the postoperative week, with comparable pain levels in NB and FB groups. Similarly, the proportion of patients reporting intake of analgesics was highest on T1 and declined steadily over the postoperative period. Intake of analgesics was more common in the FB group compared to the NB group.

The mean postoperative mouth opening for all patients on T1 was 27.2 mm, compared to 38.7mm at baseline, corresponding to an average absolute reduction by 11.2 mm and a relative reduction of 28.3%. The reduction in mouth opening was larger in the FB group compared to the NB group. The mean PoSSe score was 27.5 in the FB group and 22.0 in the NB group. More than 80% of patients reported that they had taken antibiotics and approximately 20% of patients had taken steroids after the surgery, which was much more common in the FB group than the NB group.

#### Reliability assessment

Overall, 101 complete PoSSe questionnaires (44 in the NB group and 57 in the FB group) were included in this analysis (Table 2). The reliability assessment showed a reliable Cronbach’s alpha (*α*) coefficient in all groups (*α* = 0.80 for the whole sample and also for the FB group; *α* = 0.81 for the NB group). Overall, the omission of any of the items from the PoSSe scale resulted in very minor changes to the Cronbach’s alpha; for most items this was either no change or a minor decrease, while omission of items relating to sensation and the first item relating to sickness resulted in a minor increase.

**Table 2:** Cronbach’s Alpha (*α*) coefficient of the final version of the PoSSe scale and corresponding *α* if item deleted.

Items	Overall (*n* = 101)	No bone removal (*n* = 44)	Bone removal (*n* = 57)
Overall	0.80	0.81	0.80
Eating1	0.78	0.79	0.77
Eating2	0.78	0.80	0.77
Speech1	0.79	0.80	0.79
Speech2	0.79	0.79	0.79
Sensation1	0.81	0.83	0.79
Sensation2	0.81	0.83	0.81
Appearance1	0.79	0.82	0.77
Appearance2	0.79	0.80	0.78
Pain1	0.77	0.79	0.77
Pain2	0.80	0.81	0.80
Sickness1	0.81	0.82	0.80
Sickness2	0.80	0.81	0.80
Interference1	0.77	0.79	0.77
Interference2	0.78	0.80	0.77
Interference3	0.78	0.80	0.77

#### Validity assessment

There was a positive correlation between PoSSe score and postoperative pain on each follow-up day as well as the mean pain score over the postoperative 7 days (Table 3). The overall PoSSe score was more strongly correlated with postoperative pain scores in patients who did not have bone removal compared to those who required bone removal (e.g., *r*_*s*_ = 0.73 vs. *r*_*s*_ = 0.38 for the correlation between mean pain score and PoSSe score in patients without and with bone removal, respectively).

**Table 3:** The correlation analysis between PoSSe score and postoperative pain, trismus, and surgery duration.

Analyses	N**	Overall	*n*	No bone removal	*n*	Bone removal
**Postoperative pain score (VAS)**						
T1 vs. PoSSe score	97	0.36*	41	0.59*	56	0.18
T2 vs. PoSSe score	97	0.50*	41	0.73*	56	0.36*
T3 vs. PoSSe score	97	0.51*	41	0.66*	56	0.41*
T4 vs. PoSSe score	97	0.54*	41	0.61*	56	0.45*
T4 vs. PoSSe score	97	0.45*	41	0.55*	56	0.35*
T6 vs. PoSSe score	97	0.41*	41	0.54*	56	0.26
T7 vs. PoSSe score	97	0.39*	41	0.46*	56	0.31*
Mean vs. PoSSe score	97	0.52*	41	0.73*	56	0.38*
**Trismus on T1**						
Absolute mouth opening	86	−0.37*	37	−0.38*	49	−0.30*
Absolute reduction	83	−0.30*	37	−0.10	46	−0.36*
Relative reduction	83	−0.31*	37	−0.17	46	−0.32*
**Surgery duration**	101	0.43*	44	0.31*	57	0.33*

*p <0.05.

**The number of patients varies due to missing values.

Furthermore, there was a negative association between PoSSe score and mouth opening, i.e., more severe trismus was correlated with poorer quality of life (Table 3). The corresponding correlation coefficients ranged from −0.30 to −0.37 for the various operationalisations of trismus. The correlation was stronger in patients with bone removal, while no statistically significant correlation was observed in patients without bone removal.

Additionally, the results showed that longer surgery duration was correlated with worse quality of life scores (*r*_*s*_ = 0.43) (Table 3).

## Discussion

In this study, we developed a Chinese version of the PoSSe scale and undertook a rigorous cross-cultural adaptation process with Chinese patients undergoing third molar surgery. Participants’ feedback in a pilot study documented the relevance and ease of understanding of the adapted PoSSe scale. The results from the main study indicated very good instrument performance in terms of internal consistency and validity, which can therefore be recommended for the use in clinical and research applications in Chinese patient populations. In particular, the version presented here allows future informative studies in China, also in terms of comparison across countries involving China that could assess the cultural equivalence of the measure. Our work addresses an important gap in the literature, as the PoSSe scale has already been used in China but without the necessary step of cultural adaptation [6–8].

One limitation of the presented work is that the study was conducted in a single northern municipality of China (Tianjin). Considering the varieties of Chinese dialects in different regions of China, we used the standard, unified, written Chinese (Simplified Chinese), minimising the impact of dialects on the interpretation of the scale. However, future research to confirm or otherwise its equivalent performance in other Chinese populations should be considered. Although limited in geographic coverage and size, the study sample included a typical third molar surgery patient population, including a range of clinical scenarios in terms of surgical complexity, seniority of operators, wound care, and perioperative management. Additionally, the factor structure of the original scale was never confirmed, and the measure was used as a whole score in previous applications. While the purpose of this study was to validate the existing measure for use in China, future work should evaluate the psychometric properties of the PoSSe scale more comprehensively and determine the potential domains of the scalethrough exploratory and then confirmatory factor analyses in different contexts, using datasets from different settings and populations.

Overall, the PoSSe scale is reliable and performed very well for internal consistency, with a Cronbach’s *α* coefficient well above minimum recommended standards for group comparisons [19]. Furthermore, no substantial change in the *α* value is observed when deleting any one item from the tested scale. While this would not lead to any changes to the content of the measure at this stage, future work may explore if the PoSSe scale could be shortened without significant loss of information while maintaining these high reliability ratings. In this context it is interesting to note that the omission of the items relating to impaired sensation resulted in very small increases in alpha. Neurosensory impairment represents a rare but very significant complication of lower third molar surgery but may not be a key perception of patients in terms of the immediate postoperative wound healing.

The PoSSe scale showed very good validity with significant associations in the expected directions with the extent of surgical trauma (osteotomy and duration of surgery) as well as with clinically assessed trismus and self-reported pain. It is interesting to note that the correlation of the PoSSe score with pain is much stronger in patients who did not require bone removal than those who did. One possible explanation is that while pain is an important component of postoperative quality of life - other factors such as trismus and swelling may become relatively more relevant as surgical trauma increases and may be appropriately captured by the scale.

Finally, it should be noted that we did not assess responsiveness/sensitivity to change as this is not applicable to the PoSSe scale. Unlike other PROMs that measure health status at different points in time and should be responsive to evaluate relevant changes in that health status over time or in response to an intervention, the PoSSe scale is a measure of patient perceptions just after LM3 surgery and therefore only has relevance in that specific context.

## Conclusion

The PoSSe scale has been successfully cross-culturally adapted for postoperative use among Chinese patients undergoing third molar surgery and demonstrated successful psychometric assessment for reliability and validity. The scale can be employed in future studies in relevant patient populations in China.

## Supplementary information

Supplementary tables and materials

## Data Availability

All data generated and analysed during this study are included in the manuscript and supplementary materials.
