# Supplementary material for: The cross-cultural adaptation and psychometric evaluation of a Chinese version of the postoperative symptom severity (PoSSe) scale
Source: BDJ Open. 2025 May 22;11:50. doi: 10.1038/s41405-025-00333-9 (PMC12098723; doi:10.1038/s41405-025-00333-9)
Supplement: Supplementary file 1 — Supplementary tables and materials [file 41405_2025_333_MOESM1_ESM.pdf]

Supplementary table 1. Forward Translation Report (FTR)

No.	Domains-Question		Original content	Forward draft 1	Forward draft 2	Reasons
1	Eating	a	Enjoyment	饮食方面的享受	进食	Missing enjoyment
2	Speech	b	On the worst day	在过去一周症状最严重时	——	Missing the worst day
3	Appearance	a	Bruised	淤青	淤血	Inaccurate translation
4	Eating	a	Very much	非常影响	影响很重	Difference in language expressions
5	Eating	b	How many days	（...症状）出现了多少天	（...症状）持续了多久	
6	Speech	a				
7	Appearance	a				
8	Appearance	b				
9	Pain	a				
10	Pain	a	Controlled mostly but still discomfort	基本上能控制，但仍有一些不适	服用后基本缓解，但仍有不适	Difference between literal and free translation
11	Pain	b	Poorly controlled	控制效果欠佳	服用后并无明显缓解	
12	Pain	b	Not controlled at all	完全没有得到控制	完全无缓解	
13	Interference	a	Prevent	阻止	影响	

Supplementary materials.1 Forward version of the PoSSe scale (Chinese Version)

术后生活质量调查量表

1.饮食

1）在过去的一周内，手术是否影响到了您饮食方面的享受?

A.不是，完全没有影响　　B.是的，有一点影响　　C.是的，非常影响

2）在过去的一周内，由于手术造成您不能正常开口的天数有几天?

A.0 天　　B.1-2 天　　C.3-4 天　　D.5-6 天　　E.7 天

2.言语

3）在过去的一周内，由于手术造成您的发音受到影响的天数有几天?

A.0 天　　B.1-2 天　　C.3-4 天　　D.5-6 天　　E.7 天

4）在过去的一周内，手术对您说话造成的影响最严重的一天，影响程度有多大?

A.完全没有影响　　B.轻微影响　　C.中度影响　D.严重影响　　E.完全不能说话

3.感觉

5）想想在过去的一周，有多少天您的嘴唇或舌头由于手术而感到刺痛?

A.完全没有刺痛　　B.1-2 天　　C.3-4 天　　D.5-6 天　　E.7 天

6）想想在过去的一周，有多少天您的嘴唇或舌头由于手术而感到麻木?

A.完全没有麻木　　B.1-2 天　　C.3-4 天　　D.5-6 天　　E.7 天

4.外观

7）想想在过去的一周，有多少天您的面部和（或）颈部由于手术而出现淤青?

A.完全没有青肿，瘀斑　　B.1-2 天　　C.3-4 天　　D.5-6 天　　E.7 天

8）有多少天您的面部和（或）颈部由于手术而出现肿胀?

A.完全没有肿胀　　B.1-2 天　　C.3-4 天　　D.5-6 天　　E.7 天

5.疼痛

9）想想在过去的一周，手术造成的疼痛出现了几天?

A.完全没有疼痛　　B.1-2 天　　C.3-4 天　　D.5-6 天　　E.7 天

10）想想在过去的一周，服用止痛药是否能够控制手术造成的疼痛?

A.感觉没有疼痛

B.是的，完全能控制

C.基本上能控制，但仍有一些不适

D.控制效果欠佳

E.完全没有得到控制

6.不适症状

11）想想在过去的一周，有多少天出现了呕吐症状或者感到恶心?

A.完全没有　B.1-2 天　C.3-4 天　D.5-6 天　E.7 天

12）想想在过去的一周，呕吐或者感到恶心的症状出现了多少次?

A.完全没有　B.1 天　C.2-3 次　D.超过 3 次　E.一直想要呕吐或者感到恶心

7.对日常活动的影响

13）在过去的一周内，手术是否阻止了你进行工作/家务和其他日常活动?

A.完全没有影响

B.我能继续工作，但工作受到了影响

C.是的，有 1 天不能进行工作/家务和其他日常活动

D.是的，有 2-6 天不能进行工作/家务和其他日常活动

E.是的，7 天都不能进行工作/家务和其他日常活动

14）在过去的一周内，你的业余活动是否受到了手术的影响?（包括运动，业余爱好，社会生活）

A.完全没有受到手术影响

B.轻微受到手术影响

C.中度受到手术影响

D.严重受到手术影响

15）想想在过去的一周，疼痛对你的生活产生的影响程度?

A.完全没有影响　B.轻微影响　C.中度影响　D.严重影响

Supplementary table 2. Backward Translation Report (BTR)

No.	Domains-Questions		Backward draft 1	Backward draft 2	Reasons
1	Eating	a	dietary enjoyment	diet	Missing enjoyment
2	Eating	b	speak	open your mouth	Wrong translation in the draft 1
3	Eating	a	impact	influence	Inaccurate vocabulary
4	Sensation	title	feeling	sense	
5	Sensation	a	felt stinging	been tingling	Synonym or antonym
6	Pain	a	occurred	appear	
7	Sickness	b	had	experience	
8	Pain	b	out of control	——	Missing item
9	Speech	b	What is the extent of the most serious impact of the operation on your speech in the past week	In the past week, how great its influence is when the surgery has the worst effect on your speech	Difference in language expressions
10	Pain	b	Whether taking painkillers can control the pain caused by surgery in the past week.	Did taking painkillers control pain caused by surgery in the past week?	
11	Interference	b	A. Not affected by surgery at all	A. No, it has no influence at all	
12			B. Slightly affected by surgery	B. It has slightly been affected by surgery	
13			C. Moderately affected by surgery	C. It has moderately been affected by surgery	
14			D. Severely affected by surgery	D. It has severely been affected by surgery	

Supplementary materials.2 Backward version of the PoSSe scale

Postoperative quality of life questionnaire

1. Diet
1) In the past week, has the surgery affected your dietary enjoyment?
A. No, it has no impact at all B. Yes, it has a little impact C. Yes, it has a great impact

2) In the past week, how many days have you been unable to open your mouth normally due to surgery?
A. 0 days B. 1-2 days C. 3-4 days D. 5-6 days E. 7 days

2. Speech
3) In the past week, how many days have your pronunciation been affected by surgery?
A. 0 days B. 1-2 days C. 3-4 days D. 5-6 days E. 7 days

4)What is the extent of the most serious impact of the operation on your speech in the past week?
A. No impact at all B. Slight impact C. Moderate impact D. Serious impact E. Cannot speak at all

3. Sense
5) Think about how many days in the past week have your lips or tongue felt stinging due to surgery?A. No sting at all B. 1-2 days C. 3-4 days D. 5-6 days E. 7 days

6) Think about how many days in the past week have your lips or tongue become numb due to surgery?
A. No numbness at all B. 1-2 days C. 3-4 days D. 5-6 days E. 7 days

4. Appearance
7) Think about how many days in the past week have your face and (or) neck been bruised due to surgery? A. There are no bruises and bruises B.1-2 days C.3-4 days D.5-6 days E.7 days

8) How many days have your face and / or neck become swollen due to surgery? A. No swelling at all B. 1-2 days C. 3-4 days D. 5-6 days E. 7 days

5. Pain
9) Think about how many days in the past week has the pain caused by surgery occurred?
A. No pain at all B. 1-2 days C. 3-4 days D. 5-6 days E. 7 days

10) Think about whether taking painkillers can control the pain caused by surgery in the past week.
A. I feel no pain
B. Yes, I can completely control
C. I can basically control it, but there are still some discomforts
D. The control is not good
E. Not controlled at all

6. Discomfort
11) Think about how many days in the past week have you experienced vomiting or nausea?
A. None at all B. 1-2 days C. 3-4 days D. 5-6 days E. 7 days

12) Think about how many times have you experienced vomiting or nausea in the past week?
A. No at all B. 1 day C. 2-3 times D. More than 3 times E. Always want to vomit or feel nauseous

7. Impact on daily activities
13) Has surgery prevented you from doing work / housework and other daily activities in the past week?
A. No impact at all
B. I can continue to work, but my work is affected
C. Yes, I cannot work / housework and other daily activities for 1 day
D. Yes, I cannot work / housework and 2-6 days Other daily activities
E. Yes, work / housework and other daily activities cannot be performed for 7 days

14) Has your spare time been affected by surgery during the past week? (Including sports, hobbies, social life)
A. Not affected by surgery at all
B. Slightly affected by surgery
C. Moderately affected by surgery
D. Severely affected by surgery

15) Think about how much has the pain affected your life in the past week?
A. No impact at all B. Slight impact C. Moderate impact D. Serious impact

Supplementary table 3a. Comparisons between forward version, backward version, and the original scale

No.	Domains-Questions		Original content	Forward version	Backward version	Reasons
1	Interference	title	interference	影响	impact	Translation difference caused by free translation in forward process
2	Sickness	title	sickness	不适症状	discomfort	Translation difference caused by free translation in forward process
3	Sensation	title	sensation	感觉	sense	Inaccurate selection of vocabulary in the backward translation
4	Interference	c	how badly	(影响程度)多严重	how much	Difference in language expressions/phrasing
5	Speech	b	On the worst day of the last week, how badly was your speech affected by your operation?	在过去的一周内，手术对您说话造成的影响最严重的一天，影响程度有多大?	What is the extent of the most serious impact of the operation on your speech in the past week?	Difference in language expressions/phrasing
6	Eating	a	very much	非常（地）	great	Synonym
7	Speech	b	unable	不能	cannot	Synonym
8	Sensation	a	tingling	刺痛感	stinging	Synonym
9	Sickness	b	all the time	一直	always	Synonym
10	Interference	b	leisure activities	业余活动	spare time	Synonym
11	Whole scale		thinking of	想一想/回忆一下	think about	Synonym
12	Whole scale		because of	由于	due to	Synonym
13	Whole scale		last week	过去一周的，上一周的	past week	Synonym
14	Whole scale		Severely	严重地	seriously	Synonym
15	Whole scale		operation	手术	surgery	Synonym
16	Whole scale		The simple past tense	——	The present prefect tense	Difference in the tense
17	Interference	b	prevented any social life at all	影响一切社会活动	——	Missing item in the backward translation

Supplementary table 3b. Comparisons between forward version, backward version, and the original scale

No.	Domains-Questions		Original content	Forward version	Backward version	Reasons and solutions
1	Sickness	b	On the worse day of the last week, how many times did you vomit or feel nauseated?	呕吐或者感到恶心的症状出现了多少次?	how many times have you experienced vomiting or nausea in the past week?	Missing "On the worse day", corrected in the forward version
2	Eating	title	Eating	饮食	diet	Inaccurate forward translation, corrected in the forward version
3	Speech	a	voice	发音	pronunciation	Inaccurate forward translation, corrected by regarding voice as a noun
4	Eating	a	enjoyment of food	饮食享受	dietary enjoyment	Free translation in the forward translation, linguistic improvement made

Supplementary materials.3 Preliminary version of the PoSSe scale for pilot testing

术后生活质量调查量表

1.进食

1）在过去的一周内，手术是否影响到了您进食上的享受?

A.不是，完全没有影响 [0]　B.是的，有一点影响 [5.25]　C.是的，非常影响 [10.5]

2）在过去的一周内，由于手术造成您不能正常开口的天数有几天?

A.0 天 [0]　B.1-2 天 [2.63]　C.3-4 天 [5.25]　D.5-6 天 [7.88]　E.7 天 [10.5]

2.言语

3）在过去的一周内，由于手术造成您的嗓音（声音）受到影响的天数有几天?

A.0 天 [0]　B.1-2 天 [1.25]　C.3-4 天 [2.5]　D.5-6 天 [3.75]　E.7 天 [5]

4）在过去的一周内，手术对您说话造成的影响最严重时为何种程度?

A.完全没有影响 [0]

B.轻微影响 [1.25]

C.中度影响 [2.5]

D.严重影响 [3.75]

E.完全不能说话 [5]

3.感觉

5）想想在过去的一周，有多少天您的嘴唇或舌头由于手术而感到发麻和刺痛?

A.完全没有麻刺感 [0]　B.1-2 天 [2]　C.3-4 天 [4]　D.5-6 天 [6]　E.7 天 [8]

6）想想在过去的一周，有多少天您的嘴唇或舌头由于手术而感到麻木?

A.完全没有麻木 [0]　B.1-2 天 [2]　C.3-4 天 [4]　D.5-6 天 [6]　E.7 天 [8]

4.外观

7）想想在过去的一周，有多少天您的面部和（或）颈部由于手术而出现淤青?

A.完全没有青肿，瘀斑 [0]　B.1-2 天[1.5]　C.3-4 天 [3]　D.5-6 天 [4.5]　E.7 天 [6]

8）有多少天您的面部和（或）颈部由于手术而出现肿胀?

A.完全没有肿胀 [0]　B.1-2 天[1.5]　C.3-4 天 [3]　D.5-6 天 [4.5]　E.7 天 [6]

5.疼痛

9）想想在过去的一周，手术造成的疼痛出现了几天?

A.完全没有疼痛[0]　B.1-2 天[2.38]　C.3-4 天[4.75]　D.5-6 天 [7.13]　E.7 天 [9.5]

10）想想在过去的一周，服用止痛药是否能够控制手术造成的疼痛?

A.感觉没有疼痛 [0]

B.是的，完全能控制 [2.38]

C.基本上能控制，但仍有一些不适 [4.75]

D.控制效果很差 [7.13]

E.完全没有得到控制 [9.5]

6.不适症状

11）想想在过去的一周，有多少天出现了呕吐症状或者感到恶心?

A.完全没有 [0]　B.1-2 天[1.25]　C.3-4 天[2.5]　D.5-6 天 [3.75]　E.7 天 [5]

12）想想在过去的一周，症状较严重时，呕吐或者感到恶心的次数?

A.完全没有 [0]　B.有 1 天[1.25]　C.2-3 次[2.5]　D.超过 3 次[3.75]　E.一直想要呕吐或者感到恶心[5]

7.对日常活动的影响

13）在过去的一周内，手术是否妨碍了你开展工作/从事家务和其他日常活动?

A.完全没有影响 [0]

B.我能继续工作，但工作受到了影响 [0.83]

C.是的，有 1 天不能开展工作/从事家务和其他日常活动 [1.65]

D.是的，有 2-6 天不能开展工作/从事家务和其他日常活动 [2.48]

E.是的，7 天都不能开展工作/从事家务和其他日常活动 [3.3]

14）在过去的一周内，你的业余活动是否受到了手术的影响? （包括运动，业余爱好，社会生活）

A.完全没有受到影响 [0]

B.轻微受到影响 [0.83]

C.中度受到影响 [1.65]

D.严重受到影响 [2.48]

E.手术完全妨碍了我参与所有社会活动 [3.3]

15）想想在过去的一周，疼痛对你的生活产生的影响程度?

A.完全没有影响[0]　B.轻微影响 [1.1]　C.中度影响 [2.2]　D.严重影响 [3.3]
